# The Influence of Precipitated Particles on the Grain Size in Cold-Rolled Al–Mn Alloy Foils upon Annealing at 100–550 °C

**DOI:** 10.3390/ma17071671

**Published:** 2024-04-05

**Authors:** Jianzhu Wang, Kunyuan Gao, Xiangyuan Xiong, Yue Zhang, Yusheng Ding, Jingtao Wang, Xiaolan Wu, Shengping Wen, Hui Huang, Wu Wei, Li Rong, Zuoren Nie, Dejing Zhou

**Affiliations:** 1 College of Materials Science and Engineering, Key Laboratory of Advanced Functional Materials, Education Ministry of China, Beijing University of Technology, Beijing 100124, Chinaxiangyuanxiong@bjut.edu.cn (X.X.); weiwu@bjut.edu.cn (W.W.);; 2 Yin Bang Clad Material Co., Ltd., Jiangsu Key Laboratory for Clad Materials, Wuxi 214145, China

**Keywords:** Al–Mn alloys, recrystallization, grain size, particle stimulated nucleation, Zener drag

## Abstract

The Al–Mn alloy heat exchanger fin production process includes a brazing treatment at s high temperature of 600 °C, in which coarse grains are preferred for their high resistance to deformation at elevated temperatures by decreasing the grain boundary sliding. In this study, Al-1.57Mn-1.57Zn-0.58Si-0.17Fe alloy foils cold rolled by 81.7% (1.1 mm in thickness) and 96.5% (0.21 mm in thickness) were annealed at 100–550 °C for 1 h to investigate their recrystallization behavior, grain sizes, and precipitates by increasing the annealing temperature, using micro-hardness measurement, electron back-scattered diffraction (EBSD), scanning electron microscopy (SEM), and transmission electron microscopy (TEM) techniques. The micro-hardness results showed that the recrystallization finishing temperatures for the two samples were almost the same, 323 ± 2 °C. The EBSD results showed that when the annealing temperature decreased from 550 to 400 °C, the recrystallized grain sizes of the two samples were nearly identical—both increased slightly. Further decreasing the annealing temperature from 400 to 330 °C caused the grain sizes to increase more, with the thinner foil sample having a more significant increase. The SEM and TEM observations showed that the micron-sized primary-phase remained unchanged during the annealing process. The nano-sized secondary phase precipitates formed during the hot-rolling process experienced a coarsening and dissolving process upon annealing. The particle size of the secondary phase increased from 32 nm to 44 nm and the area fraction decreased from 4.2% to 3.8%. The nucleation analysis confirmed that the large primary-phase could act as a nucleation site through particle stimulated nucleation (PSN) mode. The relatively dense secondary phase precipitates with small sizes at lower temperatures could provide higher Zener drag to the grain boundaries, leading to fewer nuclei and thereafter coarser grains. The coarsening of the recrystallized grains in the foils could be implemented through thickness reduction and/or precipitation processes to form densely distributed nano-sized precipitates.

## 1. Introduction

Al–Mn alloys have been widely used as a heat exchanger fin material owing to their high melting point, good strength, and excellent corrosion resistance. With the increasing demand for weight reduction in the automobile industry, the plate fin has been made thinner and thinner. However, it was found that the thin fins could be deformed by their own weight during the brazing process at around 600 °C due to the loss of strength [1,2,3,4,5]. Therefore, the resistance to high temperature deformation of the materials is the most important mechanical property. Coarse grains are preferred for their high resistance to deformation at elevated temperatures. The recrystallized alloy with coarse grains exhibits better deformation resistance due to its smaller creep deformation of the grain boundary [6,7,8]. Therefore, grain size is one of the key factors for improving deformation resistance, which is closely related to the annealing process of aluminum alloys in the cold-rolled state. It is generally accepted that the higher the annealing temperature, the coarser the recrystallized grains [9,10,11,12].

In addition, the recrystallized grain size is affected by the primary-phase particles and the second-phase particles present in the alloy, as these particle sizes and volume fractions play an important role in controlling the recrystallized grain size [13,14,15,16,17,18,19,20,21,22,23,24,25,26,27]. On the one hand, the coarse particles with sizes larger than 1μm could be formed during the solidification, around which the deformation zones stored sufficient energy could be formed upon (thermo-)mechanical processing, serving as nucleation sites of recrystallization. This particle stimulated nucleation (PSN) accelerates the recrystallization process by increasing the nucleation rate, which is unfavorable for forming coarse recrystallized grains [13,14,15,16]. On the other hand, the finely dispersed particles resulted from the precipitation of supersaturated solute atoms during heat treatment or thermomechanical process could provide a Zener drag to both high and low angle grain boundaries. The Zener drag from these fine dispersoids opposes the driving force of recrystallization [17,18,19], so the nucleation of the recrystallization and grain growth could be retarded and even suppressed completely [20,21,22]. 

Previous studies on the Al–Mn alloys, which were in a supersaturated state, showed complex effects of the interaction between the precipitation of fine Mn-containing precipitates and recrystallization during the annealing treatment. The kinetics of the precipitation were affected by the cold-rolled microstructure. When annealing temperature was low, significant precipitation occurred before recrystallization, and the precipitates interfered with recovery and recrystallization, decreasing the nucleation rate of recrystallization and hence leading to coarse grains after recrystallization. At high annealing temperatures, recrystallization occurred before precipitation, and the subsequently precipitated particles inhibited the process of recrystallization and grain growth, causing fine grains to form [23,24,25,26,27]. Therefore, the effect of the annealing temperature on the recrystallized grain sizes remains open. For the industrial Al–Mn alloy foils used for the heat exchanger fins, which are subjected to hot rolling and then cold rolling to an accurate thickness, the precipitation of fine Mn-containing particles can occur during hot rolling. The information on how this second-phase precipitate affects the recrystallization and recrystallized grain sizes in the alloy foils after the subsequent annealing treatment is essential for tailoring the foil properties. However, there are very few reports on the dependence of recrystallized grain size on the precipitates for this practical Al–Mn alloy. It is yet to be confirmed whether coarse recrystallized grains could be obtained in this Al–Mn alloy. 

In this paper, the recrystallization behavior and recrystallized grain sizes of Al–Mn alloy foils with different thicknesses were studied systematically using micro-hardness tests and electron microscopy techniques, including scanning electron microscopy (SEM), transmission electron microscopy (TEM), and electron back-scattered diffraction (EBSD). The primary-phase particles and second-phase precipitates during annealing at 100–550 °C for 1 h were analyzed, and their effects on the recrystallized grain sizes were discussed through PSN mode and Zener drag mechanism.

## 2. Material and Methods

### 2.1. Sample Preparation

An Al–Mn alloy ingot was prepared by melting commercial pure aluminum (99.5 wt%); high purity Zn (99.99 wt%); and Al-8 wt% Mn, Al-23 wt% Si, and Al-4 wt% Zr master alloys from Northeast Light Alloy Co., Ltd., Halben, China, in a crucible furnace at 750 °C. The melt was degassed by C_2_Cl_6_, stirred and held for 20 min, then cast into an iron mold. The actual composition of the as-cast alloy analyzed by X-ray fluorescence spectrometry is shown in Table 1. Following preheating at 500 °C for 3 h, the ingot was hot rolled from a thickness of 440 mm to 6 mm with a final temperature of 300 °C, and was subsequently reduced by 87% through multi-channel cold rolling to a thickness of 1.1 mm and by 96.5% to a thickness of 0.21 mm. 

### 2.2. Experimental Methods and Data Analyses

Samples of the two different reductions were cut by wire-cutting and then annealed at 100–550 °C for 1 h, followed by water quenching to room temperature. The recrystallization behavior after annealing at 100–360 °C for 1 h was investigated using a micro-hardness test. The recrystallized grain sizes along the rolling direction (RD) as a function of the annealing temperature for the two samples were determined using electron back-scattered diffraction (EBSD). The primary-phase and second-phase particles were observed by scanning electron microscopy (SEM), transmission electron microscopy (TEM), and scanning transmission electron microscopy (STEM).

The Vickers micro-hardness measurements on the rolling surface samples, mechanically polished to a 1 μm surface finish, were performed at room temperature using a 100 g load and a 10 s dwell time. An average of 10 measurements were taken for each sample.

The SEM samples from the planes of the RD and normal direction (ND) were prepared by electropolishing the surface in an electrolyte of 10% HClO_4_ and 90% ethanol at 20–25 V DC, and the observation conducted using a Helios 400i SEM equipped with Oxford NordlysMax3 EBSD, operated at 20 kV. 

The TEM and STEM samples were prepared by mechanically polishing to a thickness of 80–100 μm, and punching into 3 mm discs, and then twin-jet electropolishing in an electrolyte consisting of 30% nitric acid and 70% methanol at 20 V DC and a temperature below −25 °C. The TEM and STEM observations were conducted in a JEOL 2010 microscope at an operating voltage of 200 kV.

For the analysis of the recrystallization, the driving force (P_D_) for recrystallization was provided by the stored energy of the cold work according to the nucleation mechanism. The deformed microstructure consisted of well-defined subgrains because of the high stacking fault energy. The stored energy could be estimated from the subgrain diameter (D) and the specific interfacial energy (γ_S_) of the low angle grain boundaries comprising the subgrain walls. The P_D_ is provided, approximately, by [28]:

P_D_ = 3γ_S_/D (1)

γ_S_ can be calculated according to the Read–Shockley equation [29],

γ_s_(θ) = γsinθ[1 − ln(sinθ)] (2)

When a new grain with a radius R grows in the deformed structure, there is an opposing force that comes from the curvature of the high angle grain boundary of a specific interfacial energy γ. The retarding pressure on the boundary provided by the Gibbs–Thomson relationship is as follows:

P_C_ = 2γ/R (3)

Meanwhile, the Zener pinning pressure P_Z_ of the nano-scale second-phase particles on the boundary migration of the nuclei is estimated using the formation of spherical particles with a random distribution, as follows:

P_z_ = 3fγ/4r (4)

where f is the area fraction of the second-phase particles and r is the radius of the second-phase particles.

## 3. Results and Discussion

### 3.1. Micro-Hardness Measurement of Alloys Annealed at 100–360 °C 

**Figure 1:**
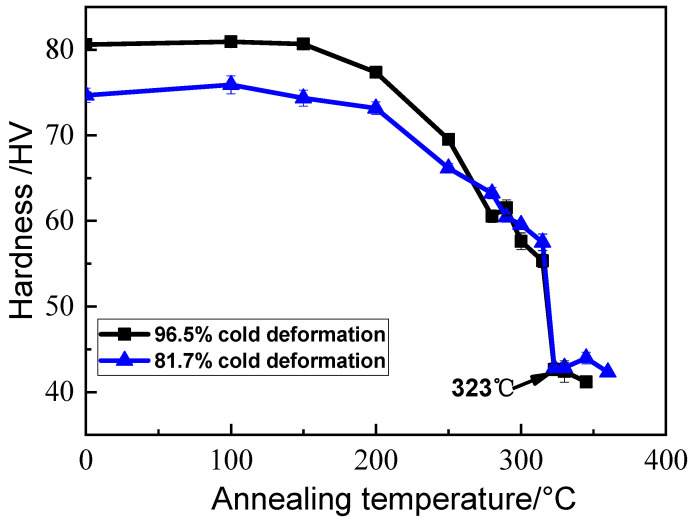
Micro-hardness changes with annealing temperature for the samples with 81.7% cold-rolling reduction (thickness of 1.1 mm) and 96.5% cold-rolling reduction (thickness of 0.21 mm). The annealing time is 1 h.

In order to determine the recrystallization finishing temperature of the two samples with 96.5% and 81.7% thickness reductions, their micro-hardness was measured as a function of the annealing temperature in the range of 100 to 360 °C for 1 h. The results are shown in Figure 1. It can be seen that with increasing the annealing temperature, the micro-hardnesses were almost unchanged at temperatures lower than 200 °C. Then, the micro-hardnesses decreased slowly between 200–315 °C, which indicates that the samples underwent recovery. The micro-hardnesses fell rapidly from 315 °C, then levelled off after about 323 °C, indicating that recrystallization occurred. Hence, the recrystallization finishing temperatures were determined to be 323 ± 2 °C for both samples, which was in good agreement with other reports [23,25]. The increase in cold-rolling reduction from 81.7% to 96.5% had no significant effect on the recrystallization finishing temperature.

### 3.2. Grain Size Change with Annealing Temperature 

**Figure 2:**
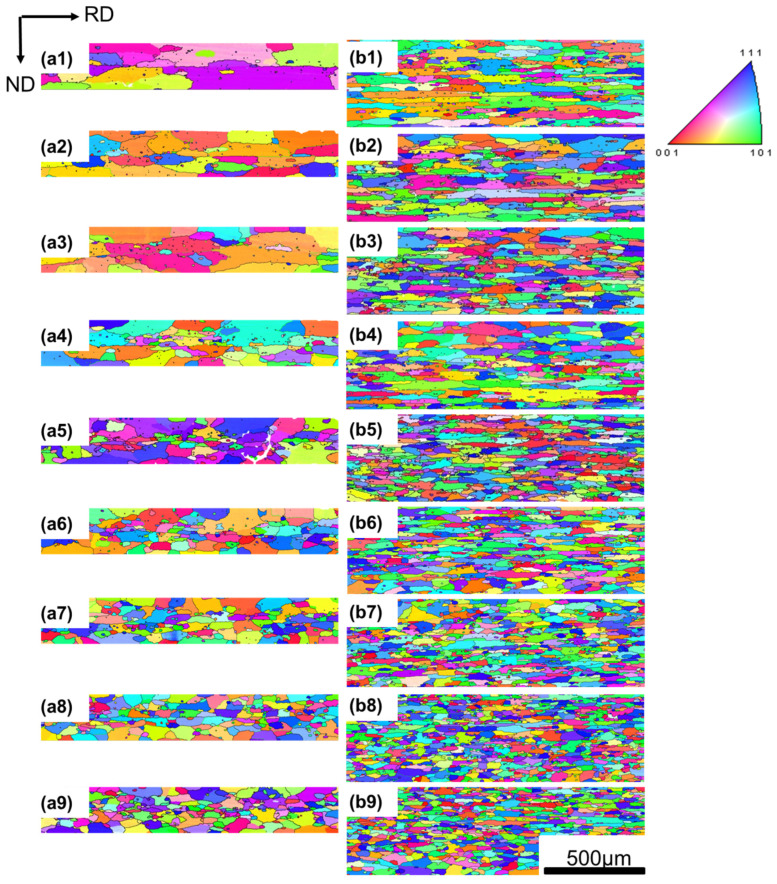
EBSD IPF maps of (**a**) 96.5% cold-rolling reduction sample and (**b**) 81.7% cold-rolling reduction sample annealed at different temperatures for 1 h: (**1**) 330 °C; (**2**) 345 °C; (**3**) 360 °C; (**4**) 380 °C; (**5**) 400 °C; (**6**) 420 °C (**7**) 450 °C; (**8**) 500 °C; (**9**) 550 °C.

The two samples with different cold-rolling reductions were recrystallized at various annealing temperatures ranging from 330 to 550 °C, i.e., higher than the recrystallization finishing temperature, to ensure that the samples were in a finished recrystallization state. Figure 2 shows the EBSD inverse pole figure (IPF) maps of the two samples annealed at various temperatures for 1 h. The EBSD images show that these samples were recrystallized completely. As seen in Figure 2(a1), there were quite a few large recrystallized grains in the 96.5% cold-rolled sample when the annealing temperature was 330 °C and the grain shape was elongated along the RD direction. With increasing the annealing temperature to 400 °C, the number of grains increased obviously and the grain size decreased along the RD and ND directions (Figure 2(a2–a5)). Further increasing the annealing temperature to 550 °C did not change the grain size much (Figure 2(a6–a9)), but the grains tended to become quasi-equiaxed. It is worth noting that the size of the grains at the surface of the sample was much larger than that of the internal grains.

Similarly, the variation in grain sizes with annealing temperature for the 81.7% cold-rolled sample showed a significant decrease in grain size in the temperature range of 330–400 °C, but remained almost the same in the temperature range of 400–550 °C, as shown in Figure 2.

Also, it was found that the grain orientations in Figure 2 were different, which may be due to the different cold reductions and different particle morphology during the annealing process.

### 3.3. Abnormal Relationship between Recrystallized Grain Sizes and Temperature

**Figure 3:**
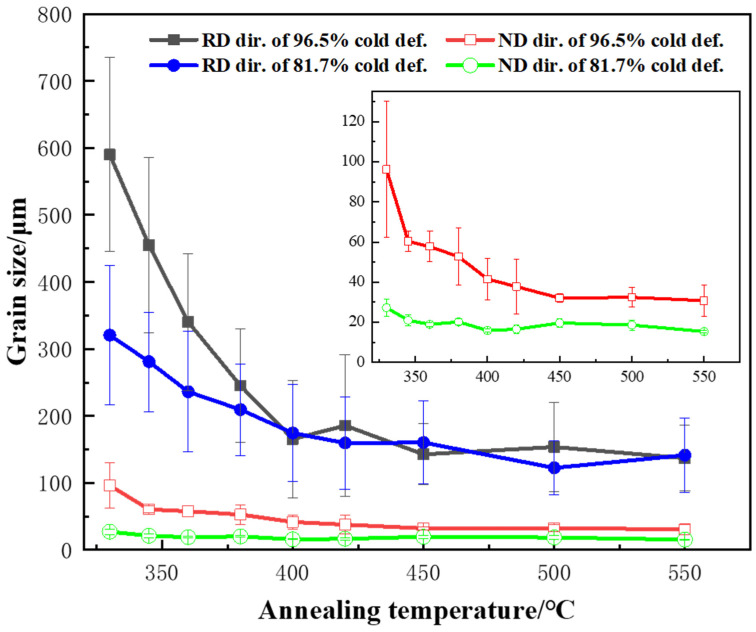
Variation of average recrystallized grain size along RD and ND direction with annealing temperature for the samples of 81.7% and 96.5% cold-rolling reductions.

To investigate the effects of the annealing temperature and sheet thickness on the recrystallized grain sizes, the grain sizes along the RD and ND directions were measured from Figure 2, and were plotted against the annealing temperature in Figure 3. As seen in Figure 3, the grain size in the RD direction far exceeded the grain size in ND direction, but their changes with the increase in annealing temperature were basically the same. With increasing the annealing temperature from 330 °C to 550 °C, the grain size along the RD decreased by 72% for the 96.5% reduction and 46% for the 81.7% reduction in the range of 330–400 °C, and then levelled off. For both the 81.7% and 96.5% cold-rolling reduction samples, the final grain sizes at 550 °C were almost the same, although their initial grain sizes at 330 °C were different. It seems that the larger deformation (the thinner thickness) gave rise to a larger change in grain size. As the recrystallization finishing temperature was 323 ± 2 °C, the samples annealed at 330–550 °C for 1 h had already been recrystallized by different extents of grain growth. The time for grain growth was longer at higher annealing temperatures, because recrystallization is a kinetic process. Apparently, this variation in grain size with annealing temperature was opposite to the general concept; that is, the higher the annealing temperature, the larger the size of the recrystallized grains. 

Similar results were reported in the supersaturated Al–Mn alloys, in which the precipitation and recrystallization were two competitive processes during the annealing treatment [23,24,25,26,27]. But the industrial Al–Mn alloys, like the one used in this study, were different, they underwent hot rolling with large deformation in the temperature range of 300–550 °C, which was the right temperature range for the precipitation process. As a result, during the subsequent cold-rolling and annealing process, recrystallization was dominating and there was no competitive process between precipitation and recrystallization. 

### 3.4. Microstructural Characterization of the Precipitates before and after Annealing Treatment 

**Figure 4:**
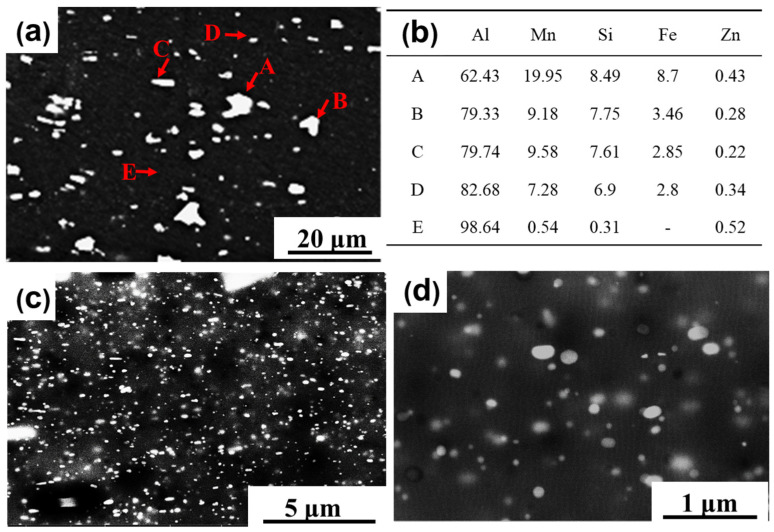
SEM micrographs and corresponding EDS of the 96.5% cold-rolled samples: (**a**) the low magnification SEM image; (**b**) the EDS analysis of the particles A–D and the matrix E in (**a**) (in at. %); (**c**,**d**) the higher magnification SEM images.

In order to clarify the above abnormal change in grain sizes with annealing temperature, the microstructures of the precipitated phase and recrystallized grains were examined in detail. The sample with 96.5% cold-rolling reduction that showed the largest decrease in grain size was selected in order to examine its microstructure before and after the annealing treatment using SEM and TEM. Figure 4 shows the SEM images at various magnifications and the local composition results obtained from the energy dispersive X-ray spectrometry (EDS). As shown in Figure 4a, a large number of coarse primary-phase particles with sizes between 0.2 μm to 5.4 μm were observed in the form of thick sticks with an irregular shape. The compositions of these primary-phase particles mainly consisted of Al–Mn–Fe–Si, as shown in Figure 4b. In Figure 4c,d, the images are shown at higher magnifications to demonstrate densely dispersed small second-phase particles with an average diameter of 32 ± 10 nm.

**Figure 5:**
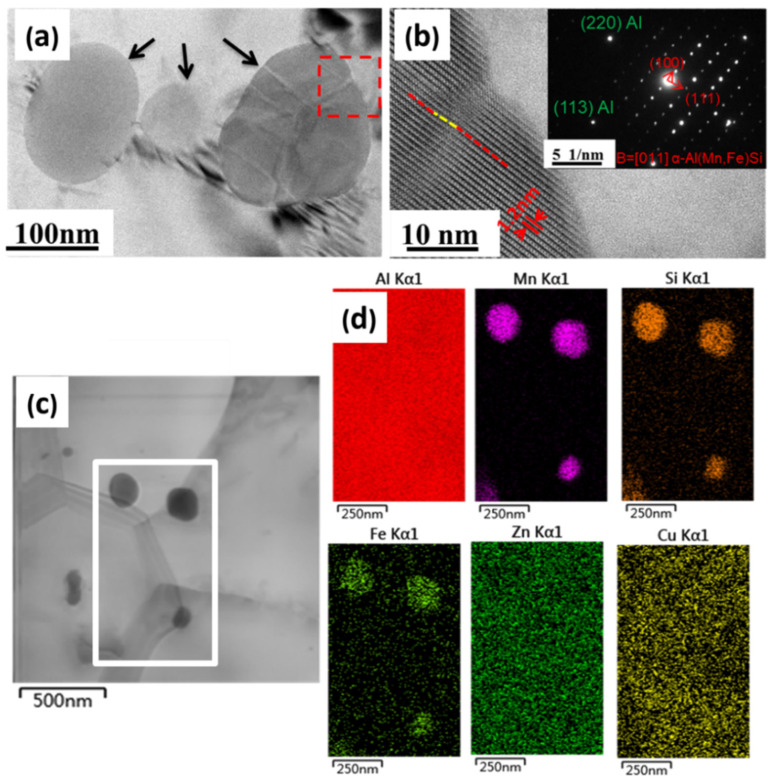
TEM micrographs and EDS mapping of the sample with 96.5% cold-rolling reduction (**a**) TEM bright field image of the precipitates; (**b**) HRTEM image and corresponding SADP of the precipitates and matrix; (**c**) TEM image; (**d**) corresponding EDS mapping of the white frame region in (**c**).

The small particles were examined using TEM. Figure 5a shows the spherical particles and the leaf-like stripes on a particle, marked with a red square. Figure 5b is a high-resolution TEM (HRTEM) image from the red square region in Figure 5a, showing a lattice image of the particle. The spacing of the lattice fringes is 1.2 nm, which corresponds to the interplanar d-spacing of plane (100) in the α-Al(Mn,Fe)Si structure [30]. The leaf-like stripes in the particle were attributed to the mismatch of atomic planes and dislocations caused by the cold rolling. A selected-area electron diffraction pattern (SADP) of the Al matrix and the precipitate in the red square in Figure 5a is shown in the inset of Figure 5b, proving that the precipitate was the α-Al(Mn,Fe)Si phase, consistent with the elemental mapping results using energy-dispersive X-ray spectroscopy (EDS) in Figure 5c,d.

**Figure 6:**
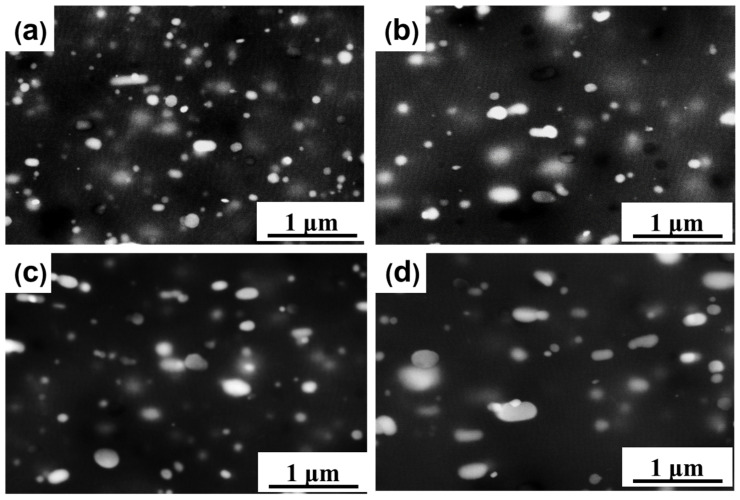
SEM micrographs of the samples annealed at different temperatures for 1 h: (**a**) 330 °C; (**b**) 360 °C; (**c**) 400 °C; (**d**) 550 °C.

The changes in the microstructure after annealing at 330–550 °C for 1 h were examined using SEM, as shown in Figure 6. With increasing the annealing temperature, the size of the fine second-phase particles increased and the number density decreased significantly. The large particles coarsened at the expense of small particles at higher temperatures. However, for the primary-phase particles, their sizes and morphology were observed to remain unchanged after the annealing treatment (not shown here).

### 3.5. The Effect of Precipitation on the Recrystallized Grain Sizes after Annealing 

According to the PSN mode, the primary-phase particles with sizes larger than 1 μm could serve as nucleation sites for recrystallization. As seen in Figure 4, there are many large primary-phase particles that are larger than 1 μm in the cold-rolled sample before annealing. These large particles could provide nucleation sites and accelerate recrystallization to form fine recrystallized grains.

**Figure 7:**
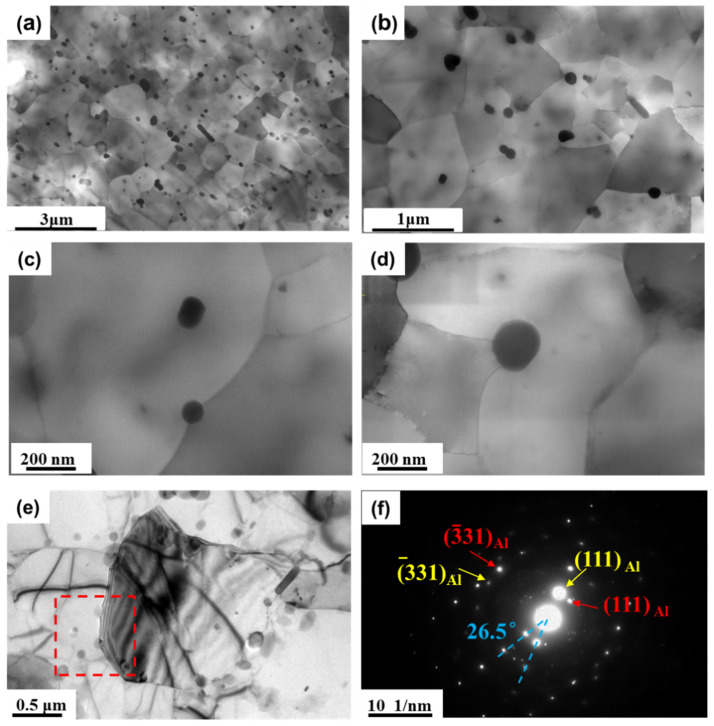
(**a**,**b**) STEM images of the 96.5% cold-rolled sample; (**c**,**d**) STEM images of the recovery sample annealed at 308 °C for 1 h; (**e**,**f**) TEM image and corresponding SADP of partially recrystallized sample annealed at 330 °C for 0.5 h.

After hot-rolling, the second-phase particles formed by precipitation were nano-scale and closely spaced. Figure 7a,b shows STEM micrographs at different magnifications for the 96.5% cold-rolled sample. There were many small precipitates distributed randomly in the matrix. These small precipitates were formed during the prior hot-rolling. It is worth mentioning that in this sample, no dispersoid-free zones were observed. 

These small precipitates could have a pinning effect (Zener drag) on both the high and low angle boundaries [17,18,19], inhibiting the nucleation and grain growth of recrystallization. Figure 7c,d shows the STEM micrographs of a partially recrystallized sample (annealed at 308 °C), in which the grain boundary was bent at the small particles. Figure 7e,f shows the boundary of the two grains and a corresponding SADP from the red square region. It can be seen that the angle between the two neighboring grains is 26.5°, suggesting that recrystallization happened. In addition, there were many precipitates at grain boundaries, which weer bent as a result of the Zener pinning effect of the precipitates.

The presence of large primary-phase particles was beneficial for the nucleation of recrystallization, and the fine second-phase precipitates inhibited the growth of the recrystallized grains. The nucleation of recrystallization was affected by the amount of deformation and the annealing temperature. 

For the calculation of the driving force (P_D_), D is 0.8 μm, estimated from the STEM images in Figure 7, and γ is the specific interfacial energy of the large angle grain boundaries, about 0.33 J/m^2^ [31]. θ is the misorientation angle of the low-angle grain boundaries, assumed to be 2°. Therefore, P_D_ is estimated to be 1.23 MPa. And for the Zener pinning pressure P_Z_, f and r can be estimated using the SEM images in Figure 6 for different annealing temperatures. 

The net driving force for recrystallization (P) is as follows:

P = P_D_ − P_Z_ − P_C_ (5)

The critical particle radius (r_g_), which allows a nucleus to grow out of the deformed zone, is therefore:

r_g_ = 2γ/(P_D_ − P_Z_) = 2γ/[P_D_ − 3γ/4(f/r)] (6)

The values of P_D_, P_Z,_ and r_g_ at different annealing temperatures are provided in Table 2. According to the calculated results, as P_Z_ decreased due to the coarsening and dissolution of the second-phase particles with the temperature, the r_g_ for PSN mode decreased continuously. 

**Figure 8:**
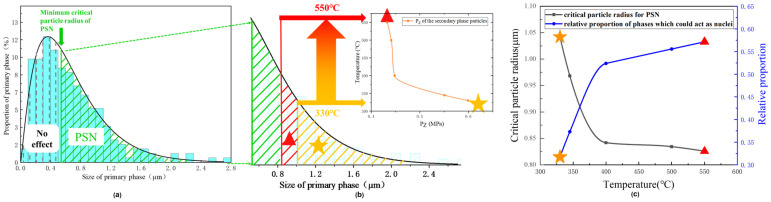
(**a**) The distribution of the primary-phase and their effect on recrystallization. (**b**) The relationship between the temperature and the critical particle radius. (**c**) The critical particle radius (black) and the percentage of primary-phase particles (blue) that could act as the nuclei as functions of the annealing temperature.

The size of the primary-phase particles was measured and their distribution was plotted, shown in Figure 8a. When P_Z_ = 0, there was no Zener pinning, and the corresponding r_g_ used the minimum value, estimated to be 0.536 μm. The primary-phase particles with a radius smaller than r_g_ had no contribution to the recrystallization, only particles greater than r_g_ could accelerate recrystallization through PSN mode.

The critical particle radii r_g_ at different annealing temperatures were marked in different colors in the size distribution diagram of the primary-phase particles in Figure 8b. With increasing the annealing temperature from 330 to 550 °C, the critical particle radius decreased, because the P_Z_ generated from the secondary phase particles decreased, as indicated by the inset of the temperature versus P_z_ curve. The decrease in the critical radius allowed more particles to act as the nuclei, increasing the nucleation rate and decreasing the recrystallized grain size.

The percentage of the total number of primary-phase particles that were large enough to act as nucleation sites was calculated, based on the particle size distribution plot. The variation in r_g_ and the percentage of large particles (greater than r_g_) with the annealing temperature are plotted in Figure 8c. As the sizes and morphology of the primary-phase particles were observed to remain unchanged after annealing, a larger percentage of effective large particles in the samples would lead to a larger amount of nucleation during recrystallization, resulting in finer grains. When the annealing temperature decreased from 550 to 400 °C, r_g_ increased slightly because P_Z_ increased. The percentage of large particles that could act as nucleation sites decreased from 31.0% to 28.5%, and the grain size became larger slightly. However, when the annealing temperature decreased from 400 to 330 °C, a sharp rise in r_g_ appeared due to the significant increase in P_Z_, because at lower temperatures, the coarsening of the particles was less significant. The percentage of large particles that could act as nucleation sites decreased from 28.5% to 17.2%, leading to a sparse distribution of nuclei and significantly coarsened grains at low temperatures, which was in good agreement with the EBSD results in Figure 2. This explains the abnormal relationship between the recrystallized grain sizes and annealing temperature observed in this study. 

As the second-phase particles in the alloy could coarsen and dissolve during the annealing treatment, different retarding effects on the recrystallization could interact with the effect of primary particles. At lower annealing temperatures, the strong Zener pinning by the fine second-phase particles caused the nucleation rate to be low, forming large grains. At higher annealing temperatures, the Zener pinning force reduced greatly and allowed more nuclei to form, resulting in small grains. This result suggests that the ratio of the area fraction to size of the precipitates needed to be reduced to obtain large grains.

From the above discussion, we conclude that when the annealing temperature is controlled at around the recrystallization finishing temperature, a coarse grain structure can be achieved for industrial Al–Mn alloy thin foils used for the heat exchanger fins.

It should be pointed out that the grain size on the sample surface was much larger than the internal grain size, as shown in Figure 2. A possible reason for this could be that the sample thickness plays an important role in the recrystallization. The surface acts as the fast diffusion channel and promotes the nucleation and grain growth greatly. This is even more the case when the temperature is lower and the foil is thinner, because a thinner foil has a larger area fraction of the surface portion in the whole thickness. More work is needed to confirm this thickness effect on the recrystallized grain size. 

## 4. Conclusions

(1) The recrystallization finishing temperature of the Al–Mn alloy cold-rolled by 81.7% and 96.5% reductions were determined to be the same, around 323 °C. When annealed at temperatures from 330 to 550 °C, the recrystallized grain size showed an abnormal variation with the annealing temperature, i.e., the lower the annealing temperature, the larger the grain size. This was more obvious in the temperature range of 330–400 °C.
(2) During the annealing process, the micro-scale primary-phase particles remained almost unchanged, providing nucleation sites for recrystallization. The coarsening and dissolution of the nano-sized secondary phase particles were observed to hinder the recrystallization process by reducing the nucleation rate and pinning the grain boundaries.
(3) The critical particle radii of PSN at different temperatures were calculated. With increasing the annealing temperature from 330 to 550 °C, the critical particle radius of PSN decreased, which increased the percentage of the effective large particles that could act as nucleation sites, leading to more grains and smaller grain sizes. In addition, the foil surface could facilitate the recrystallization process, which is more effective for the thinner foil.

## Figures and Tables

**Table 1:** Measured composition of the Al–Mn alloy.

Element	Si	Fe	Cu	Mn	Zn	Zr	Al
Content (wt%)	0.6	0.3	0.1	1.6	1.3	0.1	bal

**Table 2:** The driving pressure for recrystallization and Zener pinning pressure at different annealing temperatures.

T/°C	P_D_/MPa	P_Z_/MPa	r_g/_μm
330	1.23	0.60	1.04
345	1.23	0.55	0.97
400	1.23	0.45	0.84
500	1.23	0.44	0.83
550	1.23	0.43	0.82

## Data Availability

The original contributions presented in the study are included in the article, further inquiries can be directed to the corresponding author/s.
